# Evaluation, in a highly specialised enzyme laboratory, of a digital microfluidics platform for rapid assessment of lysosomal enzyme activity in dried blood spots

**DOI:** 10.1002/jmd2.12413

**Published:** 2024-02-19

**Authors:** Rohit Hirachan, Alistair Horman, Derek Burke, Simon Heales

**Affiliations:** ^1^ Chemical Pathology Camelia Botnar Laboratories, Great Ormond Street Hospital for Children NHS Foundation Trust London UK; ^2^ Neurometabolic Unit National Hospital for Neurology and Neurosurgery London UK

**Keywords:** digital microfluidics, dried blood spot, Fabry disease, Gaucher disease, lysosomal storage disorders, mucopolysaccharidosis I, Pompe disease

## Abstract

Lysosomal storage disorders (LSDs) are predominantly enzyme deficiencies leading to substrate accumulation, causing progressive damage to multiple organs. To date, a crucial part of diagnosing LSDs is measuring enzymatic activity in leucocytes, plasma, or dried blood spots (DBS). Here, we present results from a proof‐of‐principle study, evaluating an innovative digital microfluidics (DMF) platform, referred to as SEEKER®, that can measure the activity of the following four lysosomal enzymes from DBS: α‐L‐iduronidase (IDUA) for mucopolysaccharidosis I (MPS I), acid α‐glucosidase (GAA) for Pompe disease, β‐glucosidase (GBA) for Gaucher disease, and α‐galactosidase A (GLA) for Fabry disease. Over 900 DBS were analysed from newborns, children, and adults. DMF successfully detected known patients with MPS I, Pompe disease, and Gaucher disease, and known males with Fabry disease. This is the first demonstration of this multiplexed DMF platform for identification of patients with LSDs in a specialised diagnostic enzyme laboratory environment. We conclude that this DMF platform is relatively simple, high‐throughput, and could be readily accommodated into a specialised laboratory as a first‐tier test for MPS I, Pompe disease, and Gaucher disease for all patients, and Fabry disease for male patients only.

**Synopsis**

Digital microfluidics (DMF) could be used as a first‐tier test, in specialised diagnostic enzyme laboratories, to identify patients with mucopolysaccharidosis I (MPS I), Pompe disease, Gaucher disease, and male patients with Fabry disease.

## 1 INTRODUCTION

It has been reported that there are approximately 70 lysosomal storage disorders (LSDs).^1^, ^2^ Whilst individually, LSDs can be rare, together they may have a combined incidence of approximately 1 in 4800.^3^ LSDs arise predominantly through a deficiency of lysosomal enzymes. This leads to a toxic accumulation of biological material within the cells and tissues, causing progressive damage to organs. Clinical symptoms are heterogenous, ranging from mild to severe, and they can occur at any point from birth through to adulthood.^4^ Treatments are available for many LSDs, including enzyme replacement therapy (ERT), haematopoietic stem cell transplantation, substrate reduction therapy, chaperone therapy, and gene therapy.^5^ With therapies available and evidence showing that earlier treatment leads to more favourable outcomes for affected patients,^6^, ^7^, ^8^ there is a growing interest in detecting patients with LSDs as soon as possible.^9^, ^10^, ^11^, ^12^

A crucial part in the diagnosis of LSDs is the measurement of lysosomal enzyme activity from leucocytes, plasma, or dried blood spots (DBS). This, with genetic testing, is used to confirm a result. Increasingly, the reverse may now occur with enzymology playing a critical role in confirming a detected genetic variant. Currently, enzymology can be a complex procedure. Leucocytes must be separated from whole blood, taking approximately 45 min per sample. In our laboratory, most enzymatic assays are completed using fluorometry, and these methods typically take all day to complete (with some taking 2–3 days to generate results). During these assays, all liquid handling is performed manually by the operator. Good dexterity is required since there is repetitive pipetting, and reagents must be made and tested in the laboratory. In order to improve the efficiency of LSD testing, and the potential adoption into Newborn Screening programmes, a number of multiplex assays have been reported that utilise mass spectrometry or digital microfluidics (DMF).^13^, ^14^, ^15^ Whilst, mass spectrometry is an extremely versatile and sensitive technique, cost (the initial price and service contract), footprint, and the need for higher trained staff can limit its suitability for smaller highly specialised lysosomal enzyme laboratories. Consequently, DMF may be more acceptable to such centres.

With a view of streamlining our diagnostic processes, we have carried out a proof‐of‐principle study, evaluating a DMF platform. This platform, referred to as SEEKER®, can spatially multiplex and measure the activity of the following four lysosomal enzymes from DBS using very low volumes of reagent: α‐L‐iduronidase (IDUA) for mucopolysaccharidosis I (MPS I), acid α‐glucosidase (GAA) for Pompe disease, β‐glucosidase (GBA) for Gaucher disease, and α‐galactosidase A (GLA) for Fabry disease.^16^, ^17^, ^18^, ^19^ Individually, these enzymatic assays can be completed in less than 90 min. Furthermore, SEEKER® and the pre‐installed Spot Logic software allow the IDUA, GAA, and GBA assays to occur simultaneously. Furthermore, what makes this attractive to our Enzyme Unit, is that the chemistry of the assays is comparable to the current methods employed in our laboratory, that is, the use of 4‐methylumbelliferone substrates. Whilst these four LSDs do not overlap clinically, they are currently assessed individually in our laboratory. The aim of this study was therefore to evaluate the DMF platform, in our working environment, to ascertain whether one or more of these conditions could be more rapidly identified with regards to testing high‐risk individuals.

## 2 MATERIALS AND METHODS

### 2.1 Dried blood spot samples

Anonymised blood from a Lithium Heparin container was spotted onto a Guthrie card (Perkin Elmer 226 Ahlstrom) and left to dry overnight at room temperature. Within the same week, DBS were placed inside a sealed foil bag with desiccant and stored at −20°C. DBS were allowed to reach room temperature prior to analysis. The ages of these patients ranged from 1 day old to 89 years.

For Pompe disease testing, our laboratory already uses a DBS‐based method. For these cases, samples already arrived as a DBS or a DBS was made in the laboratory from blood as above. These DBS remained at room temperature for a prolonged period (~3 weeks) and were only stored at −20°C after analysis by our laboratory as per the standard operating procedure.

Known patients in this study had previously been identified/ had their condition confirmed by our established 4‐methylumbelliferone based assays.

For stability testing, a consenting, unaffected member of staff allowed their blood to be collected and DBS to be made.

### 2.2 Reagents and hardware

All reagents and hardware were provided by Baebies Inc. in partnership with Technopath Distribution Ltd. Four SEEKER® instruments were provided, connected to a computer and an uninterruptible power supply (UPS)—this is referred to as one workstation. The reagents provided have been described previously.^20^, ^21^

### 2.3 Protocol

Briefly, a 3.2 mm disc was punched from the back of each DBS sample into a 96‐well round bottom plate. Using a repeat pipettor, 100 μL of Extraction Buffer was added to each well containing a disc. The 96‐well plate was placed onto a plate shaker for 30 min (800 rpm at room temperature). A single‐use cartridge containing 2100 μL Filler Fluid was placed into each SEEKER®. IDUA, GAA, GBA, and GLA substrate solutions, calibrators, and Stop Buffer were allowed to thaw and reach room temperature. 3.5 μL of calibrators, 3.5 μL of each extracted DBS solution, 12 μL of each substrate solution, and 60 μL (5× 12 μL) of Stop Buffer were transferred to each cartridge, using a repeat pipettor, into the appropriate reservoirs. After loading all samples and reagents, each SEEKER® was started using the pre‐installed Spot Logic software. From here, all liquid handling and detection were automated by the workstation. From punching of the DBS to generation of the results, this process took approximately 4 h. All samples were analysed on all four SEEKER® instruments and, where the quality controls were acceptable, the average was taken to give the mean lysosomal enzyme activity. Units are ‘micromoles of 4‐methylumbelliferone (4‐MU) produced per litre of blood per hour of incubation’, or simply ‘μmol/L/h’.

### 2.4 Generation of reference ranges

Over 9 months, more than 900 samples were analysed. After this period, in‐house reference ranges were established for each enzyme using the presumed unaffected population. Initially, the results showed a log‐normal distribution. Using a Log10 transformation, the data produced an approximate normal distribution. The mean and standard deviation for each enzyme were calculated, and any results greater than the mean + 3 standard deviations were omitted (there were no results below the mean—3 standard deviations). Prior to calculating the reference ranges, 9 outliers were omitted from IDUA, 4 outliers were omitted from GAA, 9 outliers were omitted from GBA, 3 outliers were omitted from GLA in males, and 4 outliers were omitted from GLA in females. A total of 912 samples were used to create the reference range for IDUA, 928 samples were used for GAA, 883 samples were used for GBA, 500 samples were used for GLA in males, and 339 samples were used for GLA in females. The reference ranges were created on Analyse It (a Microsoft Excel add‐in), using the Parametric (log‐transformed) method.

## 3 RESULTS

Results were collected from 938 samples for MPS I (IDUA), 940 samples for Pompe disease (GAA), and 910 samples for Gaucher disease (GBA) enzymology. Since Fabry disease is an X‐linked condition, the data for males and females were separated. A total of 529 samples from male patients and 361 samples from female patients were analysed for Fabry disease (GLA). Amongst the samples, there were 5 known patients with MPS I and 12 known patients being monitored for MPS I, 8 known patients with Pompe disease, 19 known patients being monitored for Gaucher disease, 7 known males with Fabry disease and 19 known males receiving treatment for Fabry disease, and 5 known females with Fabry disease and 13 known females receiving treatment for Fabry disease. Patients being monitored for MPS I had either undergone a bone marrow transplant and were receiving ERT, or they were receiving ERT only. Six patients being monitored for Gaucher disease were receiving ERT, but there was limited information regarding treatment for the remaining 13 patients (please note that during this study, no naïve Gaucher patients were identified in the Enzyme Unit). Patients being treated for Fabry disease were receiving either chaperone therapy or gene therapy.

**FIGURE 1:**
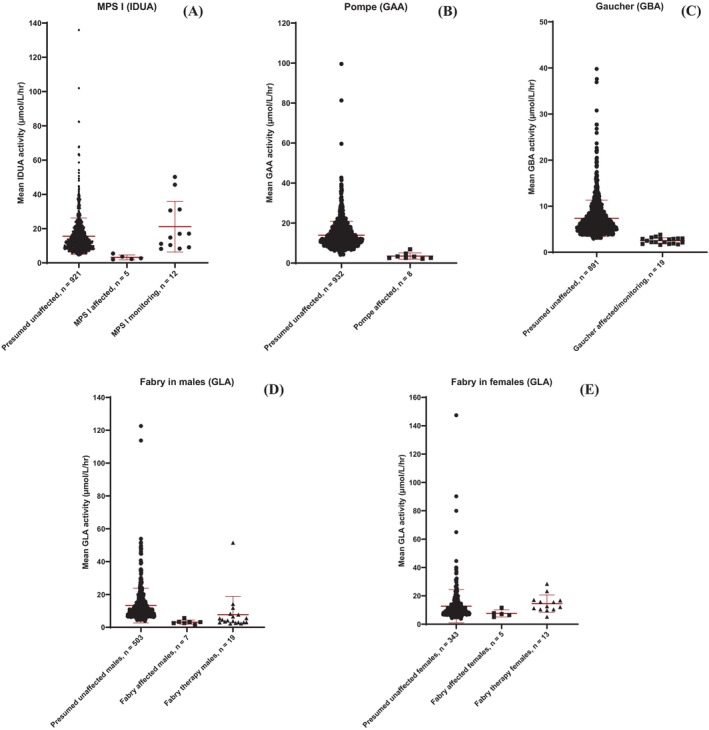
The mean lysosomal enzyme activities for (A) presumed unaffected patients, patients affected with MPS I, and patients being monitored for MPS I, (B) presumed unaffected patients and patients affected with Pompe disease, (C) presumed unaffected patients and patients being monitored for Gaucher disease, (D) presumed unaffected males, males affected with Fabry disease, and males receiving treatment for Fabry disease, and (E) presumed unaffected females, females affected with Fabry disease, and females receiving treatment for Fabry disease. Mean and standard deviations for each group are shown via the error bars. Figures were made using Graphpad Prism 10.

Figure 1A–E depicts a comparison of the lysosomal enzyme activities between presumed unaffected and known affected patients for each LSD. Activities for patients being monitored and receiving treatment can also be seen for MPS I, Gaucher disease, and Fabry disease. The mean enzyme activities for affected patients are shown in Tables 1, 2, 3, 4, 5, as well as each reference range. From these reference ranges, DMF was able to correctly identify 4 known patients with MPS I, 7 known patients with Pompe disease, 16 known patients being monitored for Gaucher disease, and 6 known males with Fabry disease.

**TABLE 1:** The mean IDUA activities for the 5 patients affected with MPS I.

MPS I affected patients	Mean IDUA activity (μmol/L/h)
Patient 1	<2.6
Patient 2	2.7
Patient 3	<2.6
Patient 4	5.5
Patient 5	3.6
IDUA reference range	5.2–33.8 μmol/L/h
Limit of detection	2.6 umol/L/h

*Note*: The reference range for IDUA was established using samples from 912 presumed unaffected patients.

**TABLE 2:** The mean GAA activities for the 8 patients affected with Pompe disease.

Pompe affected patients	Mean GAA activity (μmol/L/h)
Patient 1	4.7
Patient 2	2.6
Patient 3	2.2
Patient 4	2.6
Patient 5	2.6
Patient 6	3.2
Patient 7	6.9
Patient 8	3.3
GAA reference range	6.0–27.0 μmol/L/h
Limit of detection	1.4 μmol/L/h

*Note*: The reference range for GAA was established using samples from 928 presumed unaffected patients.

**TABLE 3:** The mean GBA activities for 6 of the 19 affected patients being monitored for Gaucher disease.

Gaucher affected/monitoring patients	Mean GBA activity (μmol/L/h)
Patient 1	<2.6
Patient 2	<2.6
Patient 3	<2.6
−−//−−	−−//−−
Patient 17	3.2
Patient 18	3.4
Patient 19	3.8
GBA reference range	3.1–13.9 μmol/L/h
Limit of detection	2.6 μmol/L/h

*Note*: For brevity, the three patients with the lowest mean GBA activities and the three patients with the highest mean GBA activities have been shown. Please note that no naïve Gaucher patients were identified during this study. The reference range for GBA was established using samples from 883 presumed unaffected patients.

**TABLE 4:** The mean GLA activities for the 7 male patients affected with Fabry disease.

Fabry affected males	Mean GLA activity (μmol/L/h)
Patient 1	<4.0
Patient 2	<4.0
Patient 3	5.6
Patient 4	<4.0
Patient 5	<4.0
Patient 6	<4.0
Patient 7	<4.0
GLA in males reference range	4.2–29.2 μmol/L/h
Limit of detection	4.0 μmol/L/h

*Note*: The reference range for GLA in males was established using samples from 500 presumed unaffected male patients.

**TABLE 5:** The mean GLA activities for the 5 female patients affected with Fabry disease.

Fabry affected females	Mean GLA activity (μmol/L/h)
Patient 1	5.1
Patient 2	11.6
Patient 3	6.4
Patient 4	7.8
Patient 5	7.1
GLA in females reference range	4.2–25.6 μmol/L/h
Limit of detection	4.0 μmol/L/h

*Note*: The reference range for GLA in females was established using samples from 339 presumed unaffected female patients.

As can be seen from Tables 1, 2, 3, 4, one known patient with MPS I, one known patient with Pompe disease, three affected patients being monitored for Gaucher disease, and one affected male with Fabry disease showed mean enzyme activities towards the lower end of the reference ranges. All five females with Fabry disease overlapped with the GLA reference range (Figure 1E, Table 5).

There were 24 presumed unaffected patients who had mean enzyme activities below the lower end of the reference ranges. This included 7 patients for IDUA, 14 patients for GAA, and 1 patient each for GBA, GLA in males, and GLA in females.

## 4 DISCUSSION

In this evaluation, the DMF platform was able to identify known patients affected with MPS I, Pompe disease, and Gaucher disease, and known males affected with Fabry disease. Furthermore, SEEKER® was able to detect known patients of various ages with severe and attenuated forms of MPS I, and patients with either Infantile Onset or Late Onset Pompe disease (IOPD or LOPD, respectively). DMF also captured patients with either non‐neuronopathic (Type 1) and neuronopathic (Type 3) forms of Gaucher disease.

One known patient with MPS I had a mean IDUA activity of 5.5 μmol/L/h which was just within the reference range (patient number 4, Table 1). This patient was 33‐years old when their IDUA activity was measured in leucocytes in our laboratory. This age is suggestive that the patient has a milder form of MPS I.

One known patient with Pompe disease had a mean GAA activity of 6.9 μmol/L/h which, again, was at the lower end of the reference range (patient number 7, Table 2). This child was 3‐years old when they were confirmed to have Pompe disease by the Enzyme Unit's fluorometric DBS and leucocyte assays. Genetic testing confirmed that the patient has late‐onset Pompe Disease (LOPD). The patient has been recorded as being active and has shown no sign of motor dysfunction, and this could be due to them having residual GAA activity. It has also been noted in the literature that some patients with LOPD could be misidentified using DBS.^22^

Since there were 19 known patients being monitored for Gaucher disease, Table 3 only shows the three lowest and three highest mean GBA activities. The three highest values all fall within the lower end of the reference range. Two of these patients had been receiving ERT and this is likely why their GBA activities are entering the range seen in presumed unaffected patients. It is not known if the third patient is receiving ERT, but they have been previously diagnosed and were being monitored prior to this evaluation.

From Table 4, one known male with Fabry disease had mean GLA activity within the reference range (patient number 3, Table 4). The request form that arrived with the sample stated that the patient does have Fabry disease. Since the diagnosis was already made, it is possible the patient was already receiving a form of treatment.

Regarding Fabry disease in females, DMF could not differentiate between known affected and presumed unaffected patients (Figure 1E, Table 5). This is not necessarily a fault with the method or technology. Due to X chromosome inactivation, Fabry disease can be incredibly difficult to determine in females through lysosomal enzyme activity alone and it has been documented that enzyme activity could potentially lead to a false negative result.^23^ Previous work in the Enzyme Unit has also shown that DBS are not as reliable as plasma and leucocytes when measuring GLA activity (data not shown). Therefore, results via DMF for Fabry disease in females should be interpreted with caution and it would be recommended to continue to measure GLA activity in both leucocytes and plasma for women suspected to have Fabry disease, as well as a genetic test.

A total of 24 presumed unaffected patients had enzyme activities below the reference ranges. A possible reason for this is that DBS specifically for Pompe disease remained at room temperature (per the laboratory's standard operating procedure) for a prolonged period (~3 weeks) before being stored at −20°C. This may have caused the activity of some of the lysosomal enzymes to deteriorate. When measuring the stability of these lysosomal enzymes, the activity of IDUA and GBA decreased by approximately 30% after 7 days when stored at room temperature (data not shown). Stability of these lysosomal enzymes in DBS has been described previously.^16^, ^17^, ^18^, ^19^ Therefore, it would be recommended that DBS be stored at 4°C or −20°C soon after they have been collected, in order to retain as much enzyme activity as possible.

Due to the nature of this study, the anonymised samples with activity below the reference ranges were not followed through. The possibility of heterozygote status, pseudodeficiencies, and variants of unknown significance cannot be excluded.

In view of potential small overlaps in patients for IDUA, GAA, GBA, and male GLA activities, we would recommend the use of additional testing (e.g., genetics and the assessment of biomarkers). Our Enzyme Unit already measures the levels of glycosaminoglycans (GAGs) in urine for patients with or suspected to have MPS I, the levels of glucose tetrasaccharide in urine for patients with Pompe disease, the activity of plasma chitotriosidase for Gaucher disease, and the levels of globotriaosylsphingosine (lyso‐Gb3) in plasma for patients affected with Fabry disease. Whilst such biomarkers can provide useful data to help achieve a definitive diagnosis, there are limitations. For example, lyso‐Gb3 is not universally elevated in Fabry disease.^24^

The primary purpose of SEEKER® is to identify newborn patients with one of the four mentioned LSDs. To our knowledge, this is the first time that this DMF platform has been used to measure lysosomal enzyme activity in affected patients who have been receiving treatment (Figure 1A, C, D, E). DMF could potentially be a rapid and convenient method of monitoring patients to see if their particular treatment is working.

The advantages and limitations of DMF have been described previously.^25^, ^26^, ^27^ In our experience, this innovative method is notably more rapid than existing assays employed in enzyme laboratories, such as ours. Furthermore, DMF requires approximately 4000‐fold less volume of sample and reagents than the methods used in our Enzyme Unit. The current assays are manual, whereas this DMF platform automates the majority of the liquid handling. Separation of leucocytes from whole blood takes approximately 45 min per sample, whereas a DBS can be collected quickly. DBS would be ideal when dealing with very young patients where it may not be possible to obtain 5–10 mL of blood. DBS are also easier to transport, and this could be of benefit when samples need to arrive from long distances. Lastly, with the existing methods, users can only analyse 8–16 samples per batch, whereas SEEKER® can accommodate 40 samples per cartridge.

## 5 CONCLUSION

Whilst SEEKER® has been used in laboratories outside of the UK,^11^, ^12^ this is the first demonstration of this DMF platform in an environment other than Newborn Screening. We conclude that this technology may have two potential uses, i.e. for use in newborn screening laboratories and to make a quick preliminary diagnosis in an urgent scenario without the need for significant manual input. More broadly, DMF is a platform that, with careful evaluation, could be extrapolated to other scenarios where rapid multiplexed assay of enzyme activity may improve patient care, and we feel that this work helps contribute to that knowledge pool.

## AUTHOR CONTRIBUTIONS

Rohit Hirachan carried out the evaluation and wrote the first draft of the manuscript. Alistair Horman, Derek Burke, and Simon Heales offered valuable input into the manuscript. Simon Heales was responsible for submitting the proposal to secure the funding to make this project possible. Simon Heales is the corresponding author. All authors reviewed and approved the submitted version of the manuscript.

## FUNDING INFORMATION

The Mucopolysaccharide Diseases (MPS Society), UK provided the funding for a Healthcare Scientist to carry out this evaluation.

## CONFLICT OF INTEREST STATEMENT

The SEEKER® instruments and reagents were loaned to the Enzyme Unit at Great Ormond Street Hospital by Baebies Inc. via Technopath Distribution Ltd., Tipperary, Ireland. The MPS Society (UK) provided an unrestricted grant to Simon Heales to cover the salary of Rohit Hirachan. Derek Burke and Alistair Horman declare that they have no conflict of interest.

## ETHICS STATEMENT

This study was a proof‐of‐principle investigation for performance evaluation of a digital microfluidics platform. Dried blood spots were created from residual patient samples that had come to the Enzyme Unit (within Chemical Pathology) for lysosomal storage disorder testing. This is in accordance with the Royal College of Pathologists (UK) Guidelines, 2012 (*Guidance on the use of clinical samples for a range of purposes that are not within the remit of Research Ethics Committees*) that permits such use of samples.

## Data Availability

De‐identified data that support the findings of this study are available from the corresponding author upon reasonable request.
